# Immunotherapies for Ischemic Stroke Patients: Current Status and Future Directions

**DOI:** 10.3390/medsci14010111

**Published:** 2026-02-25

**Authors:** Riley M. McCabe, Lauren Hurley, Kasey Pull, Nafisa M. Jadavji

**Affiliations:** 1 Department of Biomedical Sciences, Southern Illinois University, Carbondale, IL 62901, USA; 2 College of Veterinary Medicine, Midwestern University, Glendale, AZ 85308, USA; 3 Department of Biomedical Sciences, Midwestern University, Glendale, AZ 85308, USA; 4 Department of Neuroscience, Carleton University, Ottawa, ON K1S 5B6, Canada; 5 Department of Child Health, University of Arizona, Phoenix, AZ 85004, USA

**Keywords:** antibody therapy, innate immune system, ischemic stroke, neuroinflammation, sex differences

## Abstract

Background/Objectives: Ischemic stroke occurs when a thrombus forms within a blood vessel in the brain. This results in reduced levels of oxygen and glucose, leading to the death of cells and a reduction in function. There are several molecular signaling cascades that commence after the onset of an ischemic stroke; these include increases in apoptosis, oxidative stress, neuroinflammation, as well as others. The aim of this review is to provide an updated synthesis of the impact ischemic stroke has on the immune system and how the immune system can be targeted to improve outcomes post-stroke. Methods: In this review, we have included both clinical and pre-clinical studies and provide a discussion for future directions given our synthesis of the data. Results: It is evident that reducing neuroinflammation is an important component of reducing brain tissue loss and functional impairments after stroke. A combination of therapies may be more effective for stroke-affected patients. This could include reducing neuroinflammation through immune therapies, in combination with some pharmaceuticals specific to the patient’s needs. Furthermore, sex differences are highly prevalent in ischemic stroke outcomes. These differences can inform us of the best sex-specific interventions, treatments, or techniques that should be used in healthcare to ensure lower mortality rates and higher quality of life for all. Therapeutic development for ischemic stroke is needed, and this review highlights that targeting the immune system could lead to better outcomes for patients.

## 1. Introduction

Ischemic stroke occurs when a thrombus forms within a blood vessel in the brain; this results in reduced levels of oxygen and glucose to the specific area of the brain, leading to death of tissue and resulting in behavioral changes [1]. The prevalence of stroke is increasing in the population as the global population ages. Additionally, with the increased prevalence of risk factors, such as hypertension, the rate of ischemic strokes is increasing in younger populations [2].

The lack of oxygen and nutrients like glucose leads to impaired cellular function, leading to impairments in overall function. For example, if the blockage of blood flow is in the motor cortex, then motor function, such as walking, will likely be impaired in the patient. Within the brain, the anatomy of a stroke includes the ischemic core, where there is a significant loss of blood flow. The penumbra, the region adjacent to the core, has impaired function, but the structure is often preserved. Neurons and glial cells in the penumbra may be salvageable once reperfusion is established. Reperfusion is the return of blood flow after the thrombi have been removed, either spontaneously or surgically. Additionally, the process of reperfusion also contributes to damage in the brain [3].

There are several molecular signalling cascades that commence after the onset of stroke; these include increases in apoptosis, oxidative stress, and neuroinflammation [1]. In the brain, microglial cells are the resident immune cells present in all regions; they are related to monocyte and macrophage cells present in the peripheral immune system. Microglial cells are activated by various stimuli, including brain injury in pathological conditions. Activated microglial cells are the defense for the brain; they express various cytokines and growth factors in response to injury. They have two roles, first they act as phagocytes. Secondly, they release noxious substances, such as nitric oxide [4,5]. Neuroinflammation is a major contributing factor to damage after ischemic stroke and reperfusion, leading to the exacerbation of brain damage and related symptoms. Secondary health complications can also arise after the immune response is activated; this can include stroke-induced immunodepression syndrome (SIDS) and an increase in stroke severity [6]. The use of immunotherapies—including CAR-T cell therapy—may also be extendable to other conditions that predispose individuals to ischemic stroke, such as antiphospholipid syndrome [7].

Understanding the neuroinflammation response after ischemic stroke can lead to better therapeutic developments for affected patients. Inflammation prior to acute ischemic stroke (AIS) can play a significant role in outcomes [8,9,10]. Current established therapies for acute stroke include administering aspirin [11], which provides some benefit to patients, but the timing of administration is important [12]. Whereas corticosteroids have been shown to have no beneficial role for acute stroke [13]. The aim of this review is to provide an updated summary of how stroke impacts the immune system and how immune therapies can improve outcomes in both clinical and pre-clinical studies. We will also discuss future directions given our synthesis of the data.

## 2. Materials and Methods

A comprehensive literature search was conducted using PubMed to identify relevant articles published until 2024. Additional articles were identified through manual references from relevant primary articles and review papers. Only articles published in English were included. Search terms included: stroke, ischemic stroke, immune system, neuroinflammation, monocyte, immune therapy, antibodies, sex differences, sex-specific, and gene expression. Inclusion criteria were the following: studies investigating immune therapies for stroke treatment and sex differences in stroke outcomes.

## 3. Clinical Studies

In this section, we will review clinical studies that have characterized and investigated potential therapies targeting the immune system after ischemic stroke for patient recovery. This work has been summarized in Table 1.

### 3.1. Targeting Inflammation

Reducing levels of neuroinflammation might decrease damaged ischemic tissue, leading to better outcomes. Combining sodium tanshinone IIA sulfonate (STS) with tissue plasminogen recombinant activator (rt-PA) reduces cerebral hemorrhagic transformation. There is an inhibition of peripheral inflammatory cells by suppressing blood-brain barrier injury, which is critical for rt-PA effectiveness [21]. To determine the extent to which leukocytes have an effect on patient recovery, one study monitored levels of circulating monocytes in stroke patients who had received rt-PA [14]. Patients with lower monocyte counts were found to be less likely to have a poor outcome after stroke, and high monocyte counts were positively correlated with increased stroke volume and worse National Institutes of Health Stroke Scale (NIHSS) scores [14]. The NIHSS is a neurological examination that assesses the effects of stroke on consciousness, motor strength, language, and other systems that may be affected in patients [25]. However, patients who had a worse outcome were also noted to have been older with a higher NIHSS score and a larger ischemic damage volume at intake, along with other attributes that may have negatively affected their outcome [14]. Building on these findings, research has delved into the specific subtypes of monocytes that may be beneficial or detrimental. A study found that targeted modulation of CD14^high^CD16^+^ monocyte cells could increase an anti-inflammatory effect in stroke patients. This study suggests that therapies enhancing this specific subset might help minimize inflammatory damage after stroke by promoting tissue repair and angiogenesis [22].

Double-negative T cells (DNTs) were also found to be involved with post-stroke neuroinflammation. In a mixed sample study, including both 80 stroke patients and 30 mice, DNT cell levels were increased in both the brain and peripheral blood of stroke patients and mice that had undergone middle cerebral artery occlusion [26]. Furthermore, coculturing of proinflammatory CD86^+^ microglia and anti-inflammatory CD206^+^ microglia with DNT cells resulted in upregulation of proinflammatory microglia and downregulation of anti-inflammatory microglia, suggesting that DNT cells play an important role in causing increased inflammation in stroke patients [26]. Similar results were also seen in mice that were deficient in recombination activating gene 1–deficient (Rag1−/−), resulting in no mature T or B cells. These deficient mice had larger infarct volumes and worse modified neurological severity, right-turn test, and latency to fall scores after receiving DNT cells from wild-type mice [26]. One of the reasons DNT cells were thought to have such an impactful role in ischemic inflammation may be due to the release of DNT-derived tumor necrosis factor alpha (TNF-alpha), which is an inflammatory cytokine linked to activation of proinflammatory microglia. Anti-FasL antibodies were used to reduce TNF-alpha levels, resulting in reduced proinflammatory microglia activation [26]. These results indicate that anti-FasL antibodies may serve as an important treatment adjunct for patients after a stroke in reducing inflammation and infarct volume, increasing chances for an improved functional outcome.

Other studies aimed at finding treatment options that reduced leukocyte levels, thereby also reducing inflammation after ischemic stroke. One of these studies evaluated the use of intravenous minocycline, a tetracycline antibiotic that has anti-inflammatory effects by inhibiting neutrophil-mediated tissue injury, on post-stroke outcomes. Patients separated into 4 groups of incremental doses, 50% had an improved modified Rankin Scale (mRS) of 0–1, which measures the degree of disability after stroke [15]. A modified Rankin Scale score of 0 or 1 was considered an excellent outcome. However, this study did not include a control group, had a small sample size, and the results were based on a single scoring process. More extensive testing may be needed before determining clinical efficacy. Additionally, one patient’s death was potentially related to minocycline [15].

### 3.2. Supressing the Immune System

Immunosuppression may also serve as a method of reducing inflammation after an ischemic stroke in patients by hindering lymphocyte circulation into damaged tissues. Fingolimod, an immunosuppressive drug, had decreased microvascular permeability compared to the control group [16]. By decreasing microvascular permeability, the ability of lymphocytes to leave the bloodstream and circulate into tissues may be decreased. Post-stroke patients treated with Fingolimod also had limited secondary tissue injury, with ischemic damage volume extension being significantly smaller than the control group, which was assessed using T1-weighted imaging 7 days post-stroke symptom-onset [16]. This result was most likely due to lymphocytes being less able to cross blood-tissue barriers since the microvasculature was less permeable. These patients also had attenuated neurological deficits and overall better recovery, shown by an improved mean NIHSS score of 7 from 11 after one week of treatment. Higher NIHSS scores represent worse motor and neurological deficits compared to lower NIHSS scores. Even though Fingolimod proved to have positive effects on patient recovery and improvement, the sample size of this study was very small, with 11 patients in the experimental group and 11 in the control group. Additionally, the timing of drug administration was not kept consistent in this study, but the significance of this variation was not determined. To maintain consistency and accuracy, all patients in the experimental group should have been given treatment within the same time frame after admittance. Furthermore, the ratio of female to male subjects was not equally distributed, with females only accounting for about 18% of the control group and 27% of the experimental group, making the applicability of these findings to both sexes difficult [16].

Similar results were found in a study that utilized Interluekin-37 (IL-37), an immunosuppressive cytokine thought to impact stroke outcomes [17]. In this study, circulating levels of IL-37 were 2-fold higher in ischemic stroke patients within the first 3 days post-stroke; however, there was no association of this with improved NIHSS or Glasgow Coma Scale scores [17]. The Glasgow Coma Scale is a similar method to NIHSS, measuring the level of consciousness of patients with acute brain injury based on eye, motor, and verbal functions. Human subjects in this study had very few outcome measurements; therefore implications of these findings are limited since no other assessment to correlate them with other stroke outcomes was made. This study also included work in mice, and the results will be discussed in the preclinical studies section below [17].

### 3.3. Antibody Therapy

The use of antibodies to target ischemic tissue and proinflammatory cytokines was a prospective treatment in reducing ischemic infarct size and improving motor outcome of post-AIS patients in the remaining studies. Natalizumab, a monoclonal antibody against alpha4 integrin, and antibody GSK249320, a humanized monoclonal antibody to myelin-associated glycoprotein, were both investigated for this purpose. Natalizumab was used in post-AIS patients to block leukocyte-endothelium adhesion and transmigration of leukocytes into ischemic tissues, therefore reducing expansion of ischemic tissue [18]. Though both groups showed lower median infarct volumes overall, there was no significant change in medians between a placebo group of 82 patients and a Natalizumab-treated group of 79 patients, which was measured using contrasted MRI from baseline to 30 days post-treatment. Other outcomes that were assessed included effects on NIHSS, mRS, and Barthel Index (BI) scores, of which only mRS and BI scores showed improvement between placebo and treatment groups, with 13 of the 72 treated patients measured on day 30 post-treatment having perfect scores. This was not considered significant compared to the placebo group, which also had 7 placebo patients having perfect scores. There was also no significant difference between NIHSS scores from baseline to 24 h or 5 days post-treatment. Therefore, based on these results, clinical function improved in both groups, but neurological deficit did not. Overall, Natalizumab did not significantly impact infarct volume or improve clinical function scores compared to a placebo group, but additional outcome measurements could have been utilized to determine the extent of Natalizumab on circulating leukocyte counts or concentration of inflammatory markers that this drug may have affected. Lastly, there were increased infections in the treatment arm, possibly due to the non-humanised form of the antibody being used. The incidence of overall infections did not appear to be increased over the background rate [18].

GSK249320 monoclonal antibody was used in a different regard, where myelin-associated glycoprotein (MAG) was targeted to determine effects on motor outcome in post-AIS patients. MAG is an inhibitory protein that prevents outgrowth of axonal myelin, which serves as a vector for neuronal communication [19]. Targeting this protein would promote neuronal repair, improving communication and motor function. This antibody was not found to have a significant impact on gait velocity, and the study was stopped after 90 days due to results meeting a predefined futility criteria cut-off. Most subjects of both groups had some level of ambulation 180 days post-treatment, but based on other endpoints, such as gait impairment category, NIHSS score, and other mobility assessment scores, there was no significant difference between the treatment and placebo group. MAG levels were drastically decreased in patients in the GSK249320-treated group between days 1 and 6 compared to patients in the placebo group. This decrease was maintained until day 30 but returned to levels like the placebo group by day 180. Based on these findings, GSK249320 antibody treatment was not deemed a promising treatment option for motor improvement in stroke patients. Follow-up studies may be needed, as the endpoints might have had too large a floor effect, and the second antibody dosage might have been too low. This study was strong in that it utilized a larger sample population from multiple centers and countries, and patient makeup was relatively consistent in both groups, increasing diversity and confidence in their findings [19].

To target pro-inflammatory cytokines instead of glycoproteins and integrins, interluekin-1 receptor antagonists (IL-1Ra) were used to combat inflammatory responses by reducing peripheral inflammatory response and decreasing interleukin-6 (IL-6) mediator levels, which are important contributors to expansion of tissue damage in post-stroke patients [20]. IL-1Ra was found to prevent plasma IL-6 concentrations from rising in the first 3 days post-treatment compared to the placebo group. C-reactive protein concentrations were also significantly reduced in the first 3 days post-treatment, but not Von Willebrand Factor (VWF) concentrations. Decreased C-reactive protein signified a reduction in inflammation; however, VWF maintained the same levels, indicating that clot formation was still present in IL-1Ra-treated patients. Serious adverse events occurred in both experimental and placebo groups. IL-1Ra treatment was not found to have a favorable mRS outcome, and more patients in the placebo group had a mRS score of 0 to 2, meaning that motor function was not significantly different between the treatment and placebo groups. However, to further evaluate clinical outcome, this study could have also utilized additional assessment scales, such as NIHSS or Barthel Index (BI) score, since mRS was the only motor function scoring system used in this study. Overall, this study indicates that IL-1Ra may be a suitable co-treatment in post-stroke patients but requires further evaluation beyond a single center with a larger sample size, thorough safety investigation, and use of more motor and neurological function scoring systems [20].

## 4. Preclinical Studies

Preclinical studies using model systems have been used to understand and characterize the mechanisms of the immune response after ischemic stroke. Below, we will summarize the main findings from several studies, and we have also summarized each study, including the study question, model system, stroke model, and main findings in Table 2. Throughout this section, we will mention the middle cerebral artery occlusion (MCAO) model of ischemic stroke, which is an animal stroke model in which the middle cerebral artery is occluded. After some time, the occlusion is removed to mimic reperfusion, as experienced by human stroke patients [27].

### 4.1. Targeting Neuroinflammation Post-Stroke

After the onset of an ischemic stroke and subsequent reperfusion, activated leukocytes mediate neuroinflammatory responses, which is essential for removal of cellular debris. However, when the leukocytes are overactivated it can lead to the deterioration of salvageable penumbra [3,54]. One study has demonstrated that the neuroinflammatory response after an ischemic stroke can be reduced by plasmacytoid dendritic cells through microglia and regulatory T cells [28]. Using exosomes secreted from lipopolysaccharide (LPS) stimulated macrophages, neuroprotective effects were observed in vitro and in vivo stroke models. In brain tissue, ischemic damage volume was reduced, and neurological function was improved. The mechanism governing this was enhancing the microglial polarization from M1, inflammatory to M2 anti-inflammatory phenotype, thus decreasing inflammation [29]. Long-term studies are needed to identify whether these effects are maintained. Additionally, an analysis of functional outcome of animals would have provided more comprehensive data from this study.

The construction of a multifunctional nanoparticle (NP) improved MCAO-induced brain injury in male rats. The NP contained stroke-homing and blood–brain barrier targeting peptides as well as salvianic acid A, which responds to reactive oxygen species (ROS). ROS serve as signaling molecules and polarize microglia into the M1 phenotype. The M1 phenotype increases the permeability of the blood–brain barrier and allows increased leukocyte penetration as well as inflammatory cytokine secretion. Because the NP was able to eliminate ROS, regulation of microglia transformation was promoted, upholding the blood–brain barrier. The improved integrity of the blood–brain barrier led to reduced cerebral infarction, neuroinflammation, and apoptosis of neuronal cells. Improved motor and sensory function were also observed upon prolonged NP administration, though the NPs were found to distribute to a variety of metabolic organs after accumulation in the brain. Further investigation is needed to determine whether the residual NP has the potential for long-term toxicity [36].

Targeting the chemokine receptor 2 (CCR2) has provided some controversial results. Blocking the CCR2 on monocytes using a receptor antagonist, INCB 3344, worsened stroke outcome and ischemic damage volume in the brain tissue of animals after ischemic stroke, possibly because of increasing M1 polarized microglia [55]. Another study that induced stroke in CCR2 knockout mice demonstrated anti-inflammatory effects, which resulted in anti-inflammatory microglial cells and better functional outcome after stroke [56]. Manipulating the N-methyl-D-aspartate (NMDA) receptor with adeno-associated virus has also been demonstrated to have beneficial effects after ischemic stroke [33].

Chemokine-like factor 1 (CKLF1) triggers an inflammatory response of microglia which leads to the microglia entering a tolerant state and no longer responding appropriately. This decreases immune response and exacerbates ischemic stroke via the AMPK/mTOR pathway. In adult male mice, a decreased oxygen consumption rate was observed when CKLF1 was administered 1 hour before ischemia. Motor function improved when CKLF1 was inhibited by gene knockdown and antibody neutralization. Future studies may further investigate the potential for CKLF1 inhibition as a therapeutic technique [39]. The post-stroke dementia described in some patients may be mediated by B-lymphocyte infiltration [32]. As discussed above, mice deficient in T and B cells (*Rag1*^−/−^) demonstrated better stroke outcomes after day 1, including less ischemic damage volume in brain tissue and better functional scores. These measurements took place a few days after stroke. Long-term studies are needed for a better understanding of these effects [26,34]. When the current treatment for ischemic stroke, rt-PA, was combined with RTL 1000 there was no additional neuroprotective effect observed, however, the study did not include functional outcome. Transplantation of lymphocytes after ischemic stroke has been reported to decrease the number of apoptotic cells in brain tissue as well as improve functional outcome after stroke [35].

In male rats, physical exercise (PE) post-stroke can promote the expression of triggering receptors expressed on myeloid cells 2 (TREM2). Increased levels of TREM2 can be associated with long-term recovery of neurological function due to its promoting the expression of activin-A, an important component in PE-induced oligodendrogenesis [38].

Though the involvement of interleukins (IL) has been described in the clinical population, to better understand their role in ischemic stroke, model systems have been investigated. Mice transgenic for human IL-37 had an 8-fold higher level of plasma IL-37 24 h post-stroke compared to control animals, which were induced under anesthesia with exposure of the right carotid bifurcation but did not receive the filament used for MCAO in the stroke surgical mice [17]. This meant that both groups of mice were transgenic for human IL-37, but only the group that had undergone stroke had elevated plasma IL-37 concentrations. Increased expression of IL-37 mRNA had a strong negative correlation with clinical score post-stroke in transgenic mice, but actual plasma IL-37 protein did not. This was thought to be consistent with locally produced IL-37 mRNA in the brain having greater neuroprotective capabilities than circulating IL-37 protein. There were also no significant effects of IL-37 on regional cerebral blood flow or motor function, but they did find that transgenic mice travelled 2.5 times further and had a 2-fold higher maximum speed than wild-type mice [17]. Even though the difference in motor function was not significant, there was still evidence of mice having less severe locomotor deficits if able to produce IL-37. The role of IL-1 is a bit more complicated, in that there are two forms, A and B. When both are knocked out, there is a protective effect on ischemic damage volume compared to controls or single knockout animals [44]. This study only included male animals, so it would be beneficial to examine the role and impact of IL-1 in females with ischemic stroke. To reduce inflammation after a stroke, IL-4 has been administered as it has anti-inflammatory properties. Data from 1 and 7 days showed increased levels of anti-inflammatory markers, but neurodegeneration and myelin damage were still present. More long-term studies, as well as functional outcomes in animals, should be measured [46].

Cornuside, a molecule which inhibits the IL-17F/TRAF6/NF- κB pathway, offers another potential treatment for stroke-affected patients. In 8-week-old male Sprague-Dawley rats, post-stroke cornuside administration was linked to a decrease in the expression of IL-1, which maintained the integrity of the blood-brain barrier. This led to a reduction in both intestinal and neuroinflammation [51].

Intranasal administration of IL-13 was found to decrease neuron loss, exert a neuroprotective effect, and promote the integrity of white matter after ischemic stroke in adult male mice. The treatment was also able to reduce inflammation without altering the leukocyte population in the brain. The study suggests the Janus Kinase-Signal Transducer Activator (JAK-STAT) signaling pathway may be involved in the regulatory activities of IL-13, though additional research would be necessary to further investigate this [45].

### 4.2. Targeting Innate Immune System Post-Stroke

The innate immune system plays a role after ischemic stroke via TLRs. There is an increase in the expression of TLR-4 in the brain and microvasculature of animals post-stroke. Inhibiting TLR-4 after reperfusion prevents ischemic injury and improves functional outcome [48]. Two different strains of male mice were used in this study; expanding the study to include females would be beneficial. Another study showed similar findings when inhibiting TLR-4 using a monoclonal antibody, which provided decreased ischemic damage volume and reduced neurological deficits after ischemic stroke only when it was administered intra-arterially or venously [47].

Early infiltration after ischemic stroke by T-regulatory-like (TRLs) cells Cd8^+^CD122^+^CD49dlo (CD8^+^ TRLs), reduces inflammation and demonstrates neuroprotective activity in mice of both sexes. The CD8^+^ TRLs were found to infiltrate via the CXCL10/CXCR3 chemokine/receptor axis and alleviate post-stroke inflammation via production of IL-10. Epidermal growth factor-like transforming growth factor (ETGF) is present in CD8^+^ TRLs and is believed to be responsible for the neuroprotective characteristics of the cells. Intravenous treatment of CD8^+^ TRLs showed a reduction in infarction and promoted the long-term recovery in both male and female mice, posing potential for clinical implementation [40].

Cottonseed oil (CSO) has demonstrated some neuroprotective effects in adult male rats via the toll-like receptor 4/nuclear factor kappa B (TLR4/NF- κB) pathway. The treatment group demonstrated a significant decrease in brain water volume as well as increased inhibition of microglia and astrocyte inflammation [37].

### 4.3. Antibody Therapies to Promote Improved Outcomes Post-Stroke

Minocycline is an antibiotic used to treat bacterial infection and has been described to impact stroke outcome. In neonatal brain injury, minocycline has been reported to decrease ischemic damage as well as reduce neurodegeneration [49]. In male rats, minocycline administered pre-stroke reduced ischemic damage, possibly through reduced proinflammatory signalling. It is important to note that there was no behavioral analysis in terms of functional outcome measured in these rats; only brain tissue was analyzed [50]. Targeting leukocytes by using antibodies against the leukocyte adhesion molecules demonstrated no protective effect after ischemic stroke [42]. Antibodies targeting myelin-associated glycoprotein after ischemic stroke reduced ischemic damage volumes in brain tissue 7 days post-operatively, though the most beneficial effect was observed 24 h after ischemic stroke [41].

Programmed death-ligand 1 (PD-L1) and programmed death-ligand 2 (PD-L2) behave pathogenically in ischemic stroke, and one study investigates the use of anti-PD-L monoclonal antibody (mAb) as therapy for MCAO. Ninety-six hours post-mAb injection, male mice performed better on behavioral assessment, had smaller areas of neurodegeneration, less spleen damage, fewer glial cells in the brain, and increased CD8^+^ TRLs. This presents a possibility for mAb treatment to be implicated in clinical stroke patients [43].

## 5. Sex Differences

A substantial body of research has provided clear evidence of the existence of sex differences in ischemic stroke risk and outcome [23,52,53,57]. It has been found that women are at a disproportionately higher risk of stroke mortality and disability than men, while men exhibit higher numbers in stroke onset [53,57]. However, we see an inverse trend in studies done in neonatal, pre-adolescent and adolescent children, with a higher prevalence of stroke and worse stroke outcomes being observed in boys [24]. This age-dependent reversal suggests complex interactions between hormonal, genetic, and developmental factors.

Clinically, a study on patients with cardioembolic stroke showed sexual dimorphism in the early immune response, gene expression in cell death, and for genes specifically expressed by neutrophils following cardioembolic stroke. Early immune responses at the ≤ 3 h timepoint in females expressed an overrepresentation of multiple interconnected pathways including TLR (Toll-like receptor), HMGB1, TREM1, PPARa/RXRa, hypoxia, renin-angiotensin, mitochondrial dysfunction, glucocorticoid receptor and cytokine signaling pathway. These pathways play a role in preconditioning, central nervous system and periphery connection after stroke, ROS generation and cytokine production. While males had a higher expression of convergent nodes on CREB, caspase-1, and ERK1/2. The ischemia triggered phosphorylation of CREB, and subsequent CRE-mediated gene expression led to the expression of neuroprotective molecules while *CASP1* played a role in the initiation of inflammation via IL-1β activation. In the cell death pathways, at the ≤ 3 h timepoint 217 molecules participated in cell death in the females while there were only 22 molecules in the males, and 18 out of the 22 molecules in males were common in both sexes. Inflammatory responses were also female specific at this timepoint. Regarding cell-specific transcriptional changes in immune cells, females predominantly exhibited neutrophil-specific genes at the ≤3 h, 5 h and 24 h timepoints, while males only showed changes at 5 h that then increased at 24 h. These variations in gene expression contribute to the sex differences seen in stroke outcomes [23].

Beyond differences in gene expression patterns, the protective mechanism of estrogen is thought to be responsible in regulating microglial activation in females. Researchers described an increase in microglia activity in brain tissue three days after the induction of ischemic stroke using the MCAO model. There were increased levels of *TNFα* and *ptgs2* (Cox-2) expression in male mice compared to females [52]. A higher immune response was also observed in aged male mice following MCAO, they had higher levels of CD8^+^ T and regulatory T cells in the brain when compared to the females [53]. T lymphocytes are increasingly being recognized as key modulators in the detrimental inflammatory and thrombogenic responses associated with ischemic stroke [55,58]. The elevated immune response seen in males suggests that they may be more susceptible to increased tissue damage and infarct size than females following stroke induction.

## 6. Conclusions

The evidence from the clinical and preclinical studies presented in this review, it is evident that modulating neuroinflammation after stroke is important. Currently, tissue-type plasminogen activator (tPA), also known as alteplase, is the only FDA approved therapeutic for patients suffering from an ischemic stroke and the therapeutic window is limited. A mechanical thrombectomy has been shown to be beneficial for patients with proximal, large vessel occlusions up to 24 hours after stroke onset [56]. Other therapeutic options for ischemic stroke patients are needed. Recent data has revealed the Tenecteplase, is another thrombolytic agent but is manufactured by recombinant DNA technology. It has been shown to lyse large vessel clots more effectively and can be administered in a single-dose intravenously [56,59]. Additionally, targeting thrombo-inflammation using the ADAMTS13/von Willebrand axis to predict outcomes in ischemic stroke patients [60].

In terms of the data and the future for ischemic stroke therapeutics, a combination of treatments is likely to be more effective based on the data [61,62]. This could include reducing neuroinflammation through immune therapy and lifestyle changes (e.g., diet), which could provide more opportunities for recovery in stroke affected patients. Our work in mouse models has shown that motor training every day after stroke promotes recovery of function [63] and in other work we have shown that increases in B-vitamins and choline post-stroke helps with increasing better stroke outcomes [64,65] when the sensorimotor cortex is damaged.

Furthermore, sex differences are highly prevalent after ischemic stroke. The utilization of both male and female animals in preclinical work and patients in clinical work is critical in the forward progression of healthcare. These differences can inform us of the best sex-specific interventions, treatments or techniques that could be used in healthcare to ensure lower mortality rates and higher quality of life for both men and women.

Stroke has a higher prevalence in aging populations [66]. It should be noted that in recent years a worldwide trend for increased incidence of ischemic strokes in younger patients has been observed [2]. In terms of aging, the immune system also undergoes significant changes as people age [67]. Understanding these changes and how they then impact stroke outcomes will provide important insights. The lack of preclinical work conducted in aged animals needs to be increased, both female and male mice need to be included [68]. Stroke is a result of several diseases coming together and understanding those complex interactions will provide more insight into effective therapeutic development.

## Figures and Tables

**Table 1:** Summary of main findings of clinical studies, including study question, population, and main findings.

1st Author and Reference Number	Study Question(s) Being Investigated	Study Population	Summary of Main Findings
Dong et al. 2020 [14]	Are peripheral monocyte counts a reliable predictor of AIS recovery prognosis in patients receiving tPA?	206 AIS patients	Monocyte counts above 0.53 × 10^9^/L were associated with poor outcome in AIS patients (71.1% specificity, 46.2% sensitivity) Clinical factors related to poor outcomes included: infarct volume, systolic pressure at admission, and FBG Positive correlation between monocyte count and NIHSS scores at admission, 24 h after AIS, and at discharge
Fagan et al. 2010 [15]	How do increasing doses of intravenous minocycline affect post-acute ischemic stroke outcomes?	60 stroke patients	Most adverse effects were mild, self-limiting, and unrelated to dose—all reversible with no dose-limiting toxicity except 1 stroke-related death Average peak minocycline concentrations were 13 μg/mL at all doses studied beyond 3 mg/kg Peak concentrations exceeded neuroprotective levels in all but 2 patients in the 3-mg/kg dose group Two patients with lower peak concentrations (<3 μg/mL) both exceeded 100 kg and received capped dose of 270 mg (effectively <3 mg/kg) Half-life of minocycline in stroke patients is approximately 24 h allowing for once-daily dosing Pharmacokinetic parameters showed linearity across studied doses High variability in volume of distribution consistent with patient body weight variation Drug demonstrated admirable safety profile despite wide peak concentration variation suggesting wide therapeutic index
Fu et al. 2014 [16]	Does the modulation of inflammatory and immune reactions with oral fingolimod have an impact on the outcome of human AIS?	22 AIS patients	Fingolimod plus standard stroke management significantly reduced secondary injury and microvascular permeability compared to standard management alone (11 patients per group) Fingolimod-treated patients enjoyed better clinical outcomes during acute phase and at 3-month follow-up Efficacy was more apparent in patients with large infarct size than those with lacunar infarcts
Zhang et al. 2019 [17]	How does ischemic stroke effect circulating blood level of IL-37 in human ischemic stroke patients and what effect does IL-37 have on stroke outcome measures in mice transgenic for human IL-37?	79 patients (Controls—24 Ischemic stroke patients—55) 147 IL-37tg mice	Plasma IL-37 abundance was ~2-fold higher in ischemic stroke patients (55 patients) compared to controls (24 patients) in first 3 days The following was observed in IL-37tg mice: Marked increases after cerebral ischemia-reperfusion: ~9-fold plasma IL-37 and ~7000-fold brain IL-37 mRNA at 24 h Less severe locomotor deficit, smaller cerebral infarcts, and reduced bacterial lung infection compared to wild-type mice They had 60% fewer pro-inflammatory microglia-macrophages in the ischemic hemisphere Showed up to 4-fold higher expression of anti-inflammatory markers compared to wild-type mice
Elkins et al. 2017 [18]	Does the use of a single 300mg dose of natalizumab intravenously significantly decrease infarct growth in post-AIS patients compared to post-AIS patients given placebo?	161 AIS patients	No difference in median infarct volume on day 30 between placebo and natalizumab groups using MRI with contrast Both groups showed lower median infarct volumes overall Change in infarct volume across other time intervals showed no significance between groups mRS and BI scores were improved with natalizumab (13 of 72 natalizumab patients vs. 7 of 81 placebo patients had perfect scores at 30 days) but difference was not significant BI scores were significantly different between natalizumab and placebo groups on day 90 post-treatment No significant difference in NIHSS scores from baseline to 24 h or day 5 SIS-16 results on days 30 and 90 were higher in natalizumab group but not significantly higher Increased infection in the natalizumab treated group possible because of the non-humanised form of the antibody
Cramer et al. 2017 [19]	Do AIS patients who received intravenous monoclonal antibody GSK249320 have better poststroke motor outcomes than those who received a placebo?	134 ischemic stroke patients	Trial ended at interim analysis due to posterior mean treatment difference at day 90 and posterior probability being lower than predefined futility cutoff of 0.70 Most subjects had some level of ambulation by day 180 but no significant difference between groups on gait impairment category, NIHSS score, and other endpoints Majority of GSK249320 patients had no serious adverse reactions with mild to moderate adverse reactions similar to placebo group Approximately 9% of treatment group patients developed antidrug antibodies against GSK249320 MAG levels were drastically decreased in GSK249320 group between days 1–6 compared to placebo MAG decrease was maintained until day 30 but returned to placebo-like levels by day 180
Smith et al. 2018 [20]	Does subcutaneous administered Interleukin-1 receptor antagonist significantly decrease Interleukin-6 inflammatory mediator concentration and reduce peripheral inflammatory response in post-acute ischemic stroke patients compared to control group?	80 ischemic stroke patients	IL-1Ra prevented plasma IL-6 concentrations from rising in first 3 days post-treatment compared to placebo and significantly reduced the plasma curve C-reactive protein concentrations were significantly reduced in first 3 days post-treatment VWF concentrations were not significantly reduced Serious adverse events occurred in both groups: 5 serious adverse events in 5 experimental patients and 8 adverse events in 7 experimental patients Hemorrhagic transformation occurred in 6 experimental patients and 4 placebo patients IL-1Ra treatment did not have favorable mRS outcome and more placebo patients had mRS score of 0–2
Jiang et al. 2017 [6]	What changes occur in the cellular immune system and circulating inflammatory markers after ischemic stroke?	96 AIS patients and 99 age matched controls	Splenic volume correlated positively with percentages of B cells, T cells, and cytotoxic T cells but negatively with Treg cells after stroke No significant difference in lymphocyte subpopulations between ACS and PCS patients after controlling NIHSS Infections were associated with splenic volume, leukocyte counts, percentage of Treg cells, and serum levels of CRP, IL-10, and IFN-γ Serum levels of CRP, IL-6, IL-10, and IL-23 were significantly correlated with lesion volume and/or NIHSS in ischemic stroke patients Changes in cellular immune system and circulating inflammatory markers may be associated with SIDS and clinical severity
Ji et al. 2017 [21]	What are the potential effects and underlying mechanisms of STS treatment of AIS in patients who received rt-PA?	42 AIS patients	STS treatment had the following impact: Reduced the trend of cerebral hemorrhagic transformation but not significantly (possibly due to small sample size) Potentially inhibit peripheral inflammatory cells in the brain after stroke by suppressing BBB injury Protects brain from immuno-inflammation and improves recovery of stroke patients
Urra et al. 2009 [22]	Can monocyte subtypes predict clinical course and prognosis in human stroke?	46 consecutive stroke patients (ischemic (n = 35) or hemorrhagic (n = 11) stroke) and 13 age-matched controls.	Each monocyte colony has different time course in acute stroke patients and is associated with distinct clinical patterns Classic CD14highCD16- monocytes were detrimental whereas minor CD16-expressing monocyte subsets were associated with favorable clinical course Monitoring monocyte subtypes could help identify individual risk of impending complications early after stroke onset Increased CD14highCD16^+^ monocytes identifies a new trait of the immune syndrome following acute stroke to minimize inflammatory consequences of acute brain damage
Stamova et al. 2014 [23]	What sexually dimorphic immune responses do gene expressions show in peripheral white blood cells following cardioembolic stroke?	23 cardioembolic stroke patients and 23 controls without symptomatic vascular diseases	More up-regulated than down-regulated genes in both sexes following cardioembolic stroke Females had more differentially expressed genes than males following cardioembolic stroke Female gene expression was associated with cell death and survival, cell-cell signaling, and inflammation Male gene expression was associated with cellular assembly, organization, and compromise Immune response pathways were over-represented at ≤3, 5, and 24 h in females but only at 24 h in males Neutrophil-specific genes were differentially expressed at 3, 5, and 24 h in females but only at 5 and 24 h in males
Golomb et al. 2009 [24]	Is there a male predominance in childhood ischemic stroke?	Final study population was 1187 children	Childhood ischemic stroke was more common in boys regardless of age, stroke subtype, or history of trauma

Abbreviations: Acute ischemic stroke (AIS), anterior circulation stroke (ACS), Barthel Index (BI), C-reactive protein (CRP), double-negative T cells (DNT), Fas ligand (FasL), fasting blood glucose (FBG), interferon gamma (IFN-γ), interleukin (IL), middle cerebral artery occlusion (MCAO), modified Rankin Scale (mRS), myelin-associated glycoprotein (MAG), National Institutes of Health Stroke Scale (NIHSS), posterior circulation stroke (PCS), sodium tanshinone IIA sulfonate (STS), stroke-induced immunodepression syndrome (SIDS), tissue plasminogen activator (tPA), tumor necrosis factor alpha (TNF-α), Stroke impact scale (SIS), Von Willebrand Factor (VWF).

**Table 2:** Summary of main findings of preclinical studies.

Author Last Name and Year of Publication	Study Question	Model System and Stroke Model Used	Main Findings
Chen et al. 2020 [28]	What is the role of pDCs in ischemic stroke?	110 male C57BL/6 mice tMCAO	MCAO significantly increased pDC percentage in the brain Antibody-induced depletion of pDCs resulted in: ○ Significantly increased infarct size ○ Elevated IL-6 and TNF-α plasma levels ○ Significantly reduced IL-10 plasma levels pDC depletion significantly reduced Treg cells levels in the brain, spleen, and blood. They were also found to prime and accelerate Treg induction
Zheng et al. 2019 [29]	What is the role of inflammation in the pathogenesis of ischemic stroke? What are the effects of administering exosomes secreted from the lipopolysaccharide -stimulated macrophage RAW264.7 cell line LPS-Ex after ischemic stroke? What is the mechanism of stroke-induced inflammation and/or the anti-inflammatory effect(s) of LPS-Ex treatment?	BV2 cells, RAW264.7 cells, and 15 Sprague Dawley rats Model of transient focal cerebral ischemia.	LPS-Ex treatment was found to do the following: Significantly down-regulate inflammatory cytokines (NF-κB p65, TNF-α, IL-6) compared to Ex alone LPS treatment induced M1 polarization with higher CD80 expression Promote M2 polarization with increased CD206 and enhanced Arg1/iNOS ratio Enhance SH-SY5Y cell viability and reduced apoptosis Demonstrate neuroprotective effects in vivo: ○ Significantly reducing infarct size ○ Significantly improving neurological scores Attenuate ischemia-induced increases in NF-κB, TNF-α, and IL-6 levels Dil-LPS-Ex penetrated the BBB and reached ischemic cerebral tissue after ischemia/reperfusion
Chu et al. 2015 [30]	What is the role of CC chemokine receptor 2 (CCR2)-dependent Ly6Chi monocytes in ischemic stroke?	102 male C57BL/6 mice Middle cerebral artery occlusion (MCAO) model	CCR2^+^ monocyte subpopulation increased and was dose-dependently reduced by INCB3344 Ly6Chi monocyte expression was inhibited by INCB3344 MCAO caused reduced hanging grip time INCB3344 treatment worsened stroke outcomes, increased infarct volume and M1-polarized macrophages
Fang et al. 2018 [31]	What is the role of CCR2 dependent MoDMs in brain injury after stroke? What is the role of CCR2-dependent MoDMs in stroke outcome/recovery?	167 male C57BL/6J WT 153 CCR2 KO mice MCAO model	CCR2 KO mice had smaller infarcts and less mortality in early post-stroke days, while in later post-stroke days, they had larger infarcts and higher mortality Propagermanium treatment showed biphasic effects: smaller infarcts at 3 days, larger infarcts at 14 days Anti-inflammatory microglia/macrophages transfer improved outcomes in CCR2 KO mice
Doyle et al. 2015 [32]	Does B-lymphocyte response to stroke contribute to the onset of dementia? Does B-cell involvement present a new treatment target for stroke?	C57BL/6 mice BALB/CJ mice muMT C57BL/6J background mice Population not specified DMCAO	B-lymphocytes infiltrated the infarct after 1-week delay and maintained high levels at 7- and 12-weeks post-injury Mice developed short-term memory deficits coincident with B-cell accumulation muMT mice did not develop memory deficits IgA levels significantly increased between weeks 1 and 7 in stroke lesions, suggesting local isotype switching LTP was impaired in the hippocampus at 7- and 12-weeks post-injury Anti-CD20 antibody treatment prevented: ○ Delayed cognitive deficits ○ B-cell and IgG recruitment to the brain Human stroke/dementia subjects had significantly higher B-lymphocyte density compared to controls with IgG in perilesional tissue
During et al. 2000 [33]	Does a NR1-subunit-targeted vaccine (AAVNMDAR1) have therapeutic potential for cases of NMDA-mediated neuronal injury? Does AAVNMDAR1 effectively cross the BBB into the immune-privileged CNS? Does a cerebral insult potentiate greater BBB/CNS permeability and facilitate fewer adverse effects?	104 Rats Species not specified Kainate model of temporal lobe epilepsy Endothelin-1 model of MCAO	A single dose of AAVNMDAR1 vaccine successfully induced transgene expression and generated antibodies for various NMDAR1 epitopes AAVNMDAR1 vaccination suppressed kainate-induced seizure activity and prevented typical seizure-induced neuropathology Infarct volume reduced by 70% in MCAO stroke model treated with AAVNMDAR1 compared to naive or AAVlac-treated animals
Kleinschnitz et al. 2010 [34]	What is the contribution of T-cells in cerebral ischemia? What are the minimal immunologic requirements for T-cell activation? What is the mechanism of action for T-cell-mediated effects: antigen-specific recognition or non-antigen-specific interactions? What involvement (if any) to T-cells have in thrombus formation?	219 multiple C57Bl/6 strains, deficient in various T-cells, B-cells, and/or cofactors (2C/*Rag2*, OT-I/*Rag1* (MHC-I), OT-II/*Rag1* (MHC-II), 2D2/*Rag1*, *Cd28*−/−, *Pdcd1lg1*−/−, *Pdcd1*−/−, *Cd1d1*−/−, *Tcrd*−/−) Population not specified tMCAO model	*Rag1*−/− mice had significantly reduced infarct volumes and better functional scores post-tMCAO B-cell reconstitution did not affect stroke protection T-cell reconstitution made deficient mice susceptible to tMCAO-induced infarcts and functional deficits TCR signaling pathways were not essential for early stroke progression Nonclassical T-cell populations (NKT and γδ) were not involved in stroke progression Reconstitution with OTI/RAG1 or 2D2/RAG1 splenocytes induced WT-like detrimental effects, confirming T-cell involvement in ischemic injury No differences in thrombus formation or stability between RAG1−/− and WT mice
Zhao et al. 2019 [35]	What are the effects of the transplantation of lymphocytes co-cultured with human cord blood-derived multipotent stem cells (HCB-SCs) on the inflammatory response in transient middle cerebral occlusion (tMCAO)-subjected rats?	34 Male Wistar rats tMCAO model	tMCAO significantly increased neurological impairment and tape removal latency at 48 h compared to sham animals A significant increase in inflammasome proteins (caspase-1, mature IL-1β, NLRP1, NLRP3) at 48 h was also observed post-stroke LT treatment did the following: significantly reduced caspase-1, mature IL-1β, NLRP3 levels, p-ERK/total ERK and p-NF-κB ratios as well as apoptotic cell numbers in the ischemic cerebral cortex Significantly reduced both functional scores and infarct/lesion size Augmented the MCAO-induced increase in CD4^+^/CD25^+^/Foxp3^+^ regulatory T cells (Tregs)
Li et al. 2024 [36]	In what ways does oxidative stress contribute to neuronal damage? What inflammatory pathways are most significant in neuronal damage after ischemic stroke? Can drugs target these issues and would this treatment be biocompatible?	31 male Sprague-Dawley rats, human neuroblastoma (SH-SY5Y) and HBMECs MCAO/R	Stepwise multi-targeted treatment using ORD@SHp@ANG nanoparticles significantly improved ischemic stroke recovery Multifunctional nanoparticles consisted of: ○ Stroke homing peptide (DSPE-PEG2000-SHp) ○ BBB-targeting peptide (DSPE-PEG2000-ANG) ○ ROS-responsive Danshensu (salvianic acid A) chain self-assembly Treatment approach combined substances to reduce oxidative stress and suppress inflammation
Liu et al. 2020 [37]	What is the role of CSO in neuroprotection against stroke?	184 adult male rats MCAO/R model	CSO treatment significantly reduced infarct volume and neurological deficit scores compared to MCAO control groups Treatment with CSO also significantly decreased brain water content and inhibited microglial and astrocyte inflammation through TLR4/NF-κB pathway
Xu et al. 2023 [38]	Does PE promote oligodendrogenesis and remyelination? If so, how is exercise act as a neuroprotectant?	140 Male Sprague-Dawley rats tMCAO model	PE promotes TREM2 expression in rats after stroke TREM2 upregulation promotes white matter integrity and is associated with long-term neurological function recovery Microglial state and activin-A expression are regulated by TREM2 upregulation TREM2/activin-A pathway is important in PE-induced oligodendrogenesis
Ma et al. 2023 [39]	What is the role of microglia in ischemic stroke etiology? How does the relative amount of chemokine like factor 1 (CKLF1) effect the activity of microglia and the inflammatory response?	23 adult male C57BL/6N mice Photothrombosis model	CKLF1 enhances glycolytic ability of microglia In microglia CKLF1 inhibits oxidative phosphorylation pathway Glycolysis is necessary for C27-activated microglia (supported by multiple studies)
Cai et al. 2022 [40]	Through what mechanisms do CD8^+^ TRLs infiltrate the brain post-stroke? What pathways regulate the neuroprotective effects of CD8^+^ TRLs?	C57BL/6J WT, Rag1-KO, Cxcr3-KO, Cxcl10-KO, Tgfa-KO, and Il10-KO mice of both sexes Population not specified tMCAO model	CD8^+^ TRLs infiltrate the ischemic brain through the CXCL10/CXCR3 chemokine/receptor axis They were also found to alleviate poststroke inflammation via IL-10 Neuronal protection is provided by ETGF present in TRLs
Cash et al. 2016 [41]	Does administration of GSK249320 restore or improve behavioral outcomes after middle cerebral artery occlusion?	66 male Sprague Dawley rats MCAO model	All infarct volumes decreased over time but no significant differences between GSK249320 treatment groups regarding lesion size, sticky tape test, and cylinder test measures GSK249320 administered 24 h post-MCAO significantly increased neuroscores compared to vehicle at 2/4 weeks as well as pellets retrieved in staircase task compared to vehicle at 2/4/6 weeks GSK249320 staining qualitatively increased in the ipsilesional hemisphere with increasing time since MCAO
Bowes et al. 1993 [42]	Does administration of (ICAM-1 reduced reperfusion injury in CNS ischemia? Does ICAM-1 increase the therapeutic effect of tPA?	175 New Zealand white rabbits MCAO model	Delayed tPA at 30- or 90-min significantly increased clot weight needed to produce damage (abnormal behavioral scores) Anti-ICAM treatment at 30- or 90-min significantly increased clot weight needed to produce damage Combined tPA and anti-ICAM treatments produced no additional benefits compared to either treatment independently When tPA was delayed 180 min, no significant effects were observed for either treatment
Bodhankar et al. 2015 [43]	What is the role of PD-L1 and PD-L2 in stroke severity? Can monoclonal Ab (mAb) inhibit PD-L1, and will this reduce inflammation and neurodegeneration?	133 adult male Wild type C57BL/6J mice MCAO model	mAb injection resulted in the following: Improved behavioral performance at 96 h Resulted in smaller areas of neurodegeneration Reduced spleen damage Decreased glial cell numbers in the brain Increased CD8^+^ regulatory t cells
Boutin et al. 2001 [44]	What are the roles of IL-1α and IL-1β in ischemic brain damage? Does the deletion of one of the ligand-relevant genes generate a compensatory mechanism? What is the effect of IL-1 receptor agonist (IL-1ra) treatment on the IL-1 knockout mice?	C57BL/6 WT, IL-1α KO, IL-1β KO, and IL-1αβ KO mice Animal population: 42 MCAO model	Dual-KO (IL-1αβ) mice had significantly reduced total and cortical infarct volumes compared to WT or single-KO mice They also had significantly reduced edema compared to WT MCAO-driven cerebral ischemia induced IL-1β expression in both WT and IL-1α KO mice Cerebral ischemia did not induce significant changes in IL-1α or IL-1ri expression in WT animals Intracerebroventricular IL-1ra injection significantly reduced total and cortical infarct size in WT and IL-1α KO mice IL-1ra administration had no effect in IL-1β KO or IL-1αβ KO mice
Chen et al. 2022 [45]	How does IL-13 effect long-term recovery after ischemic stroke? What is its role in microglia and macrophage polarization?	Adult male C57BL/6J mice. Population not specified tMCAO model	Intranasal IL-13 administration improves neuron loss and exerts long-term neuroprotective effects IL-13 treatment promotes white matter integrity post-ischemic stroke and reduces inflammation without altering brain leukocyte populations JAK-STAT signaling pathway may be involved in IL-13 regulatory activities
Lively et al. 2016 [46]	Does early supplementation with IL-4 shift the brain towards an anti-inflammatory state and reduce damage after transient focal ischemia?	Adult male Sprague-Dawley rats Population not specified ET-1 transient focal ischemia model	24 h after ET-1-induced ischemia, ipsilateral striatum showed pro-inflammatory state with elevated TNF, NOS2/iNOS, and IL-6 IL-4/ET-1 injection increased anti-inflammatory markers at 7 days: ○ Itgam (1.6-fold increase) ○ Cd200r1 (1.8-fold increase) ○ Tlr2 (1.7-fold increase) ○ Tlr4 (1.7-fold increase) ○ Hmox1 (3.6-fold increase) IL-4 treatment had the following impact: Augmented delayed increase in CD11b-expressing microglia/macrophages Increased phagocytic microglia/macrophages (Iba1^+^, CD68^+^) at day 1 Increased neutrophil entry likely via increased VEGF-A transcript levels Increased neurodegeneration by 29% and increased astroglial response 1.7-fold in non-infarct striatal tissue
Andresen et al. 2016 [47]	Does therapeutic anti-TLR4 antibody (clone MTS510) modify TLR-4 activation, infarct volume, inflammatory cell accumulation and neurological deficits after middle cerebral artery occlusion? Which (if any) method of antibody delivery (imp, IA, or iv) minimizes detrimental effects while protecting against ischemic brain injury?	In vitro: RAW 264.7 cells (Abelson leukemia virus-transformed cell line from BALB/c mice) In vivo: 105 male C57Bl/6N wild-type mice MCAO model	Anti-TLR4/MD2 mAb treatment did the following: Intraperitoneally it showed no significant effects on infarct volume, neurological deficit, or cell numbers Intra-arterially it significantly reduced infarct volumes and improved neurological deficits Intravenously it significantly reduced direct infarct volumes and neurological deficits Increased activated macrophages and microglia in the contralateral (non-ischemic) hemisphere Increased T-cell counts in both ischemic and non-ischemic hemispheres Increased absolute B-cell numbers in the contralateral hemisphere
Abdul et al. 2018 [48]	Does inhibition of TLR-4 signaling reduce hemorrhagic transformation, and improve stroke outcome in diabetes? Does inhibition of TLR-4 signaling mediate the effects of extracellular iron in vascular endothelial cell death?	In vivo: 35 male Wistar rats (diabetes induced via intraperitoneal streptozotocin) In vitro: BMVECs isolated from spontaneously diabetic male GK rats MCAO model	Ischemic stroke increases TLR-4 expression in the brain and microvasculature of diabetic animals The following were observed in TLR-4 inhibition: After reperfusion, it prevents amplified neurovascular injury (greater infarct size, hemorrhagic transformation, excess hemoglobin) in diabetes Improves functional outcomes in diabetic animals Restores BMVEC membrane permeability and increases tight junction protein expression Increases cell survival and angiogenic potential
Arvin et al. 2002 [49]	Is systemic minocycline treatment protective against H-I brain injury? Is this neuroprotective effect of minocycline treatment dose-dependent and/or modulated by delivery pre- or post-injury? Does minocycline treatment block H-I induced caspase-3 activation in the neonatal brain? Does minocycline treatment inhibit caspase-3-like activity?	320 Sprague-Dawley litters of postnatal day 7 pups A model of hypoxia-ischemia based on the Levine procedure	Minocycline treatment prevented H-I-related percent area decrease across striatum (23.3% vs. 6.5%), hippocampus (21% vs. −4.2%), and cortex (29.3% vs. 4.3%) in a potentially dose-dependent manner The treatment animals showed no caspase-3 or substrate cleavage in cortex ipsilateral or contralateral to carotid ligation Effects of the treatment were observed when given immediately after insult but attenuated when given three hours post-insult PBS-treated animals had increased active caspase-3, PARP, and spectrin only in cortex ipsilateral to carotid ligation Vehicle-treated animals showed increased DEVD-AMC cleavage activity (six-fold in ipsilateral vs. contralateral hippocampus, eight-fold in ipsilateral vs. contralateral cortex) Minocycline given immediately before hypoxia resulted in nearly equivalent bilateral cleavage activity in hippocampus and cortex
Yrjanheikki et al. 1999 [50]	Is minocycline neuroprotective against ischemic stroke? What effects does minocycline have on cortical infarction volume prior to onset of ischemia and after onset of ischemia?	32 Male Sprague-Dawley rats Control group population not specified Neuronal cultures from Wistar rat pups Intraluminal suture MCAO model– Longa method	The following occurred with minocycline treatment: 12 h pre-stroke it reduced infarct size by 76% in cerebral cortex and 39% in striatum 2 h post-ischemic injury it reduced infarct size by 65% cortical and 42% striatal 4 h post-injury it showed 63% cortical reduction 12 h prior to ischemia, it reduced CD11b-immunoreactive cells associated with inhibition of microglial activation and cytotoxic effects Inhibited ICE enzyme expression responsible for IL-1β activation Minocycline pretreatment reduced PGE2 concentrations by 55% in the penumbra No significant difference in cortical spreading depression between treatment and control groups
Yan et al. 2024 [51]	Can cornuside effectively treat ischemic stroke and what are its therapeutic mechanisms?	145 eight-week-old male Sprague–Dawley rats. MCAO/R model	Ischemic stroke rat model showed increased intestinal permeability and dysbiosis of intestinal microflora Cornuside improved dysregulation of intestinal microflora and reduced intestinal inflammation and neuroinflammation by inhibiting the IL-17F/TRAF6/NF-κB pathway
Villapol et al. 2019 [52]	Do differential acute neuroinflammatory response and infiltrated immune cells occurs between male and females after three days post-ischemia?	64 male and female C57BL/6J mice pMCAo model	Increased microglia/macrophages occurred in male versus female mice with amoeboid morphology TNFα and *ptgs2* (Cox-2) genes were more expressed in males More myeloid cell infiltration was found in male versus female brains No sex-dependent differences in injured volume or cell death density were observed Sex differences in acute microglial and immune responses to neonatal ischemia are likely gene- and region-specific
Ahnstedt et al. 2020 [53]	What are the sex differences in central and peripheral T cell immune responses after stroke in aged mice? What are the sex differences in gut permeability, microbiota diversity, and behavioral outcomes after stroke in aged mice?	Aged C57BL/6CR mice Population not specified MCAO model	Mortality and hemorrhagic transformation were significantly higher in males than in females No difference in infarct size at 3 days between sexes Stroke induced greater gut permeability of FITC-dextran in males at day 3 Non-reversible alterations in microbiota diversity occurred in males Both sexes showed a time-dependent increase of CD4^+^ and CD8^+^ T cells in the brain Significantly higher levels of CD8^+^ T cells and Treg cells in males at day 15 Aged males demonstrated greater neurological deficits up to day 5 and impaired sensorimotor function up to day 15 Trend toward greater cognitive decline in males at day 21 Increased GFAP expression in both sexes at 42 days post-stroke indicating astroglial activation and gliosis

Abbreviations: Blood brain barrier (BBB), brain microvascular endothelial cells (BMVECs), central nervous system (CNS), chemokine like factor 1 (CKLF1), chemokine receptor 2 (CCR2), cottonseed oil (CSO), distal middle cerebral artery occlusion (dMCAO), endothelin 1 (ET-1), epidermal growth factor–like transforming growth factor (ETGF), fluorescein isothiocyanate (FITC), gamma delta T cells (γδ), glial fibrillary acidic protein (GFAP), Goto-Kakizaki (GK), human brain microvascular endothelial cells (HBMECs), hypoxic-ischemic (H-I), immunoglobulin A (IgA), immunoglobulin G (IgG), intracellular adhesion molecule 1 (ICAM-1), interleukin-1β converting enzyme (ICE), knockout (KO), lipopolysaccharide exosomes (LPS-Ex), long-term potentiation (LTP), lymphocyte transplant (LT), middle cerebral artery occlusion/reperfusion (MCAO/R), monoclonal antibody (mAb), monocyte-derived macrophages (MoDMs), Homozygous mutant mice that lack mature B cells (muMt), myeloid differentiation factor 2 (MD2), N-methyl-D-aspartate (NMDA), N-methyl-D-aspartate receptors (NMDAR), natural killer T cells (NKT), permanent proximal middle cerebral artery occlusion (pMCAO), phosphate buffered saline (PBS), physical exercise (PE), plasmacytoid dendritic cells (pDCs), poly(ADP-ribose) polymerase (PARP), programmed death ligand 1 (PD-L1), prostaglandin E2 (PGE2), reactive oxygen species (ROS), regulatory T cells (Tregs), T cell receptor (TCR), tissue plasminogen activator (-type), toll-like receptor (TLR), transient middle cerebral artery occlusion (tMCAO), T regulatory-like cells (TRLs), triggering receptor expressed on myeloid cells 2 (TREM2), vascular endothelial growth factor A (VEGF-A), wild type (WT).

## Data Availability

No new data were created or analyzed in this study.

## Abbreviations

The following abbreviations are used in this manuscript:

AIS	Acute ischemic stroke
ACS	Anterior circulation stroke
BBB	Blood–Brain Barrier
BI	Barthel Index
BMVECs	brain microvascular endothelial cells
CKLF1	chemokine like factor 1
CCR2	chemokine receptor 2
CNS	Central Nervous System
CRP	C-reactive protein
CSO	cottonseed oil
dMCAO	distal middle cerebral artery occlusion
ET-1	Endothelin-1
ETGF	epidermal growth factor–like transforming growth factor
DNT	Double-negative T cells
FasL	Fas ligand
FBG	Fasting blood glucose
FITC	fluorescein isothiocyanate
Γδ	gamma delta T cells
GFAP	glial fibrillary acidic protein
GK	Goto-Kakizaki
HBMECs	human brain microvascular endothelial cells
H-I	hypoxic-ischemic
ICAM-1	intracellular adhesion molecule 1
ICE	interleukin-1β converting enzyme
IFN-γ	Interferon gamma
IgA	immunoglobulin A
IgG	immunoglobulin G
IL	Interleukin
KO	knockout
LPS-Ex	lipopolysaccharide exosomes
LTP	long-term potentiation
LT	lymphocyte transplant
mAb	monoclonal antibody
MCAO	Middle Cerebral artery occulsion
MCAO/R	middle cerebral artery occlusion/reperfusion
MoDMs	monocyte-derived macrophages
mRS	modified Rankin Scale
MAG	myelin-associated glycoprotein
MD2	myeloid differentiation factor 2
muMt	Homozygous mutant mice that lack mature B cells
NMDA	N-methyl-D-aspartate
NMDAR	N-methyl-D-aspartate receptors
NHSS	National Institutes of Health Stroke Scale
NKT	natural killer T cells
PARP	poly(ADP-ribose) polymerase
PBS	phosphate buffered saline
PE	physical exercise
pDCs	plasmacytoid dendritic cells
PD-L1	programmed death ligand 1
PGE2	prostaglandin E2
pMCAO	permanent proximal middle cerebral artery occlusion
PCS	posterior circulation stroke
ROS	reactive oxygen species (ROS)
STS	sodium tanshinone IIA sulfonate
SIDS	stroke-induced immunodepression syndrome
TCR	T cell receptor
Tregs	regulatory T cells
TLR	toll-like receptor
tMCAO	transient middle cerebral artery occlusion
TNF-α	tumor necrosis factor alpha
tPA	tissue-type plasminogen activator
TRLs	T regulatory-like cells
TREM2	triggering receptor expressed on myeloid cells 2
SIS	Stroke impact scale
VEGF-A	vascular endothelial growth factor A
VWF	Von Willebrand factor
WT	wild type
